# An impairment-specific hip exoskeleton assistance for gait training in subjects with acquired brain injury: a feasibility study

**DOI:** 10.1038/s41598-022-23283-w

**Published:** 2022-11-11

**Authors:** Chiara Livolsi, Roberto Conti, Eleonora Guanziroli, Þór Friðriksson, Ásgeir Alexandersson, Kristleifur Kristjánsson, Alberto Esquenazi, Raffaele Molino Lova, Duane Romo, Francesco Giovacchini, Simona Crea, Franco Molteni, Nicola Vitiello

**Affiliations:** 1 The BioRobotics Institute, Scuola Superiore Sant’Anna, Pontedera, Pisa, Italy; 2 Department of Excellence in Robotics & AI, Scuola Superiore Sant’Anna, Pisa, Italy; 3 IUVO S.R.L., Pontedera, Pisa, Italy; 4 Villa Beretta Rehabilitation Center, Valduce Hospital, Costa Masnaga, Lecco, Italy; 5 Össur, Reykjavík, Iceland; 6 Department of PM&R, MossRehab and Einstein Healthcare Network, Elkins Park, PA USA; 7 IRCCS Fondazione Don Carlo Gnocchi ONLUS, Florence, Italy

**Keywords:** Biomedical engineering, Movement disorders

## Abstract

This study was designed to investigate the feasibility and the potential effects on walking performance of a short gait training with a novel impairment-specific hip assistance (iHA) through a bilateral active pelvis orthosis (APO) in patients with acquired brain injury (ABI). Fourteen subjects capable of independent gait and exhibiting mild-to-moderate gait deficits, due to an ABI, were enrolled. Subjects presenting *deficit in hip flexion and/or extension* were included and divided into two groups based on the presence (group A*, n* = *6*) or absence (group B, *n* = *8*) of *knee hyperextension* during stance phase of walking. Two iHA-based profiles were developed for the groups. The protocol included two overground gait training sessions using APO, and two evaluation sessions, pre and post training. Primary outcomes were pre vs. post-training walking distance and steady-state speed in the 6-min walking test. Secondary outcomes were self-selected speed, joint kinematics and kinetics, gait symmetry and forward propulsion, assessed through 3D gait analysis. Following the training, study participants significantly increased the walked distance and average steady-state speed in the 6-min walking tests, both when walking with and without the APO. The increased walked distance surpassed the minimal clinically important difference for groups A and B, (respectively, 42 and 57 m > 34 m). In group A, five out of six subjects had decreased knee hyperextension at the post-training session (on average the peak of the knee extension angle was reduced by 36%). Knee flexion during swing phase increased, by 16% and 31%, for A and B groups respectively. Two-day gait training with APO providing iHA was effective and safe in improving walking performance and knee kinematics in ABI survivors. These preliminary findings suggest that this strategy may be viable for subject-specific post-ABI gait rehabilitation.

## Introduction

Stroke and other acquired brain injuries are a leading cause of long-term disabilities, often entailing hemiparesis, sensory-motor impairment, cognitive alteration, and speech disturbances^1^. Among stroke-induced impairments, gait alteration is considered one of the major factors that negatively impact the patient’s independence and quality of life^2–4^. Therefore, recovering functional walking ability is a central goal of rehabilitation programs^5,6^.

Current therapeutic guidelines promote intensive, repetitive, task-specific mobility training^7^. Powered robots have emerged as promising tools to deliver high-dosage and task-oriented therapy without physically overloading the therapists. Various forms of exoskeletal robots target gait re-education of individuals post-ABI^8^. In clinical practice, treadmill-based robotic-assisted gait training (RAGT) in combination with body-weight support is the most common robotic rehabilitation approach and has shown positive effects on walking function^9–12^.

Previous studies have shown that simple repetitive motor activity alone is not sufficient to guide neuroplasticity (i.e. to produce a functional reorganization of cortical maps). Therefore, RAGT should not consist only of intensive and repetitive stereotyped movements in a constrained environment (such as the treadmill) but should include locomotor training following principles of motor learning such as overground walking in real-world environments (increasing task specificity, variability of practice, sensory cues and interaction with realistic environments) that promote neuroplasticity^13,14^. Training with a mobile exoskeleton during overground walking removes the constraints of a treadmill while still allowing the use of a robot to assist the patient. From this perspective, overground RAGT should be preferred over treadmill-based RAGT when possible, since it enhances trunk balance, trunk control and the contribution of the visual-spatial and vestibular components, as well as patient engagement^13^.

Full lower-limb mobile exoskeletons for overground walking have been used in several clinical studies^15–17^. However, for individuals who have unilateral involvement and retain walking capacity, such as the majority of post-stroke patients (up to 80%)^18^, these devices may not be the best approach to promote a more functional walking pattern^19^, as their weight, encumbrance and type of control may limit their usability. Light-weight lower-limb exoskeletons which selectively assist some of the lower-limb joints are emerging as promising alternative robotic tools^20–29^. These devices allow the patient to walk overground in a real-world environment, encouraging trunk and balance control and promoting a more physiological gait pattern^30,31^.

Hemiparetic gait is characterized by unilateral muscle weakness, spasticity, impaired inter-limb coordination and insufficient forward propulsion, resulting in reduced gait speed, asymmetric walking and altered kinematic patterns^32,33^. During the swing phase of walking, the movement of the hemiparetic limb is frequently characterized by limitations in hip flexion, knee flexion and ankle dorsiflexion that can result in reduced limb clearance and limb advancement with undesirable compensatory movements such as hip hiking and/or circumduction^34^. During the stance phase, decreased hip extension and ankle plantarflexion are common deficits and different types of abnormal knee patterns such as excessive knee flexion (knee buckling) or knee hyperextension can be observed. Among post-stroke patients, 40–68% present with knee hyperextension^35^. This impairment is a disabling deviation that can be progressive and affects gait speed, symmetry, and efficiency, and can generate knee pain and other joint changes^36^.

Literature reports different types of treatment to reduce knee hyperextension, such as surgical interventions^37^, orthoses [e.g. ankle–foot orthoses (AFOs), active knee orthoses (AKOs), knee-ankle–foot orthoses (KAFOs)]^38–40^, proprioceptive training^41^ and functional electrical stimulation^42^ among others. An example is the use of AFOs to mitigate knee hyperextension by manipulating plantarflexion resistance during the stance phase^43^. However, until now, there is no strong evidence of the effectiveness of any of these treatments. As suggested by Bleyenheuft^44^, the most appropriate treatment should be identified based on aetiology. Several aetiologies may explain this gait deviation (e.g. weakness or spasticity of knee extensors muscles, weakness of hip extensor or knee flexor muscles, weakness, spasticity or contractures of ankle plantar flexors, proprioceptive disorders), and it is common to observe a combination of aetiologies in the same patient^44^. Considering that during the stance phase the leg is in a closed kinetic chain, applying a phase-synchronized torque through a hip exoskeleton might be a viable strategy in some cases, still unexplored, to counteract knee hyperextension. In fact, applying an external force at the thigh segment can generate an effect on the whole kinetic chain, in particular on the joints of the involved segment (hip and knee)^45^.

Analysis of the available literature points out that overground RAGT using bilateral hip exoskeletons has the potential for improving walking performance in post-stroke individuals with mild-to-moderate gait impairments^22,25^. Still, several research questions remain, particularly on whether hip assistance may be used to improve knee kinematics and whether hip assistance can be tailored to the specific gait impairment of each patient. In previous clinical studies with stroke subjects, hip assistance was delivered as a biomimetic torque vs. gait phase profile, i.e. with a flexion torque during the swing phase and an extension torque during the stance phase. Customization of the assistance was limited to minor adjustments in timing and amplitude of the delivered assistance through a manual and iterative process under the control of the experimenter^20,22,25^, and the details on the exact timing or torque adjustment are not reported in the literature.

Based on this framework, this study intended to further investigate the use of a hip exoskeleton in post-ABI over-ground gait rehabilitation along two lines of action. Firstly, we explored the feasibility of implementing impairment-specific hip assistance (iHA), with a hip flexion torque in the stance phase for patients presenting with knee hyperextension. The working hypothesis was that a resistive flexion torque applied to the hip during the stance phase could reduce the occurrence of knee hyperextension. Secondly, we explored the potential effects of short gait training with the proposed impairment-specific hip assistance (iHA) on walking performance in individuals with mild-to-moderate gait deficits, due to an ABI. The proposed methodology was assessed by considering the impact of a short training period with APO, a bilateral powered hip orthosis, primarily on gait speed and endurance (assessed through 6MWTs), which are primary indicators of locomotor function in post-ABI rehabilitation, as they are associated with a better quality of life^46,47^, and secondarily on joint kinematics and kinetics, gait symmetry and propulsion.

## Materials and methods

### Study participants

Suitable candidates were identified as individuals capable of independent gait (with or without assistive tools) exhibiting mild-to-moderate gait deviations, due to a neurological disease. Individuals were eligible for inclusion if they met the following criteria: (1) age > 18 years old, (2) capable of independent gait with or without assistive devices, i.e. Functional Ambulation Category scale 2–4^48^; (3) demonstrable unilateral or bilateral hip weakness with concomitant gait deviation, i.e. Medical Research Council (MRC) hip strength 2–4; (4) ability to follow 3-step commands. Throughout the study duration, participants could use their ambulation assistive devices, such as canes, functional electrical stimulation (FES) and AFOs. Exclusion criteria were as follows: (1) physician disapproval for participation, (2) relevant comorbidities (e.g. chronic heart failure, uncontrolled diabetes or hypertension, chronic obstructive pulmonary disease, severe hip/knee osteoarthritis, severe osteoporosis, severe sensory deficit), (3) Modified Ashworth Scale (MAS) at the hip > 3, (4) cognitive impairment (Mini-Mental State Examination score < 21), (5) severe anxiety or depression (State-Trait Anxiety Inventory > 44, Beck Depression Inventory > 19), (6) skin wounds or infection at interaction points with the exoskeleton interface, (7) implantable cardiac devices, such as pacemakers or automatic defibrillators, (8) pelvis width outside the range [340; 410] mm.

In total fourteen subjects with ABI were enrolled for this study. Of these, thirteen subjects were chronic (time from injury > 6 months), and one was subacute (time from injury 5 months); thirteen participants had a stroke while one participant had a traumatic brain injury (age: 51.5 ± 11.8 years; four female/ten male; time since ABI: 5 ± 4 years; eight subjects exhibited right-side hemiparesis). Enrolled subjects had MRC muscle strength 2–5, and MAS 0–2 in hip and knee joints (Supplementary Table I, II).

Study participants exhibiting *deficit in hip flexion and/or extension* were included in the study and divided into two groups based on the presence (group A*, IDs* = *1–6*) or absence (group B, *IDs* = *7–14*) of *knee hyperextension* during walking.

A description of all fourteen participants is reported in Table 1. All participants provided informed consent before starting the study.

**Table 1:** Study participants’ characteristics.

ID	Side of paresis	Sex	Age (y)	Weight (kg)	Pathology	Time from injury	Orthosis or other assistive device	Baseline walking speed (m/s) and FAC	Main gait impairment identified during stance	Main gait impairment identified during swing	Group assignment
1	right	M	62	82	(isch) stroke	Chronic (10y)	none	0.68	knee hyperextension	circumduction	A
								FAC 3			
2	left	F	43	60	(haem) stroke	Chronic (8y + 2mo)	none	0.88	knee hyperextension	stiff knee	A
								FAC 4			
3	left	F	29	50	traumatic brain injury	Chronic (28 2y + 4mo)	AFO	0.40	knee hyperxtension	deficit in hip flexion	A
								FAC 3			
4	left	M	50	84	(isch) stroke	Chronic (12y)	none	0.40	knee hyperextension	deficit in hip flexion	A
								FAC 3			
5	left	M	54	70	(haem) stroke	Chronic (8mo)	AFO	0.60	knee hyperextension	deficit in hip flexion	A
								FAC 3			
6	right	M	56	82	(haem) stroke	Chronic (3y)	none	0.70	knee hyperextension	deficit in hip flexion	A
								FAC 3			
7	right	F	56	51	(isch) stroke	Chronic (3y + 2mo)	cane	0.33	deficit in hip extension	stiff knee	B
								FAC 2			
8	right	M	69	79	(isch) stroke	Chronic (3y + 9mo)	none	0.58	knee buckling	deficit in hip flexion	B
								FAC 3			
9	left	M	38	87	(isch) stroke	Chronic (2y + 10mo)	none	0.71	deficit in hip extension	deficit in hip flexion	B
								FAC 3			
10	right	M	47	70	(isch) stroke	Chronic (8 mo)	TA FES	0.55	deficit in hip extension	hip hiking	B
								FAC 3			
11	right	F	54	56	(isch) stroke	Chronic (5y + 8mo)	none	0.79	deficit in hip extension	reduced limb clearance	B
								FAC 3			
12	right	M	43	78	(haem) stroke	chronic (2y + 2mo)	none	0.39	deficit in hip extension	stiff knee	B
								FAC 2			
13	left	M	73	84	(isch) stroke	sub-acute (5mo)	none	0.40	deficit in hip extension	deficit in hip flexion	B
								FAC 2			
14	right	M	47	82	(isch) stroke	Chronic (8y)	AFO, cane	0.70	deficit in hip extension	hip hiking	B
								FAC 3			

*F* female, *M* male, *haem* haemorrhagic, *isch* ischemic, *y* years, *mo* months, *TA FES* Functional Electrical Stimulation for Tibialis Anterior, *AFO* Ankle Foot Orthosis, *m/s* meters/seconds, *FAC* Functional Ambulation Category.

### The bilateral powered hip orthosis

The Active Pelvis Orthosis is a bilateral powered robotic hip orthosis (or exoskeleton) designed to power hip flexion or extension gait movements by providing smooth assistive torque at the hip, adapting to natural gait variations. The APO can provide support to individuals who retain walking capacity with or without assistive devices and present mild-to-moderate gait impairments (FAC ≥ 2). Walking impairments can be classified by comfortable walking speed as mild (> 0.7 m/s), moderate (0.3–0.7 m/s) and severe (< 0.3 m/s)^49^. Since the APO provides neither weight support nor balance assistance, it is not intended for individuals who require assistance or body weight support to walk. The APO system used for this study was based on the same mechatronic architecture as previously-reported prototypes^50,51^, with additional design optimizations for portability and weight reduction to 6.5 kg.

From the mechatronics viewpoint, the APO is built around a carbon-fiber frame that surrounds the user’s hips and posterior pelvis, and interfaces with the trunk and hips via a customisable orthotic cuff. The APO frame carries a backpack –housing the control electronics and battery– and two actuation units, one on each side, employing a series elastic actuator architecture^52^. Each actuation unit is deployed along two parallel axes. The first axis (posteriorly located) is the output shaft of a 70-Watt-BLDC motor coupled with a 1:100 reduction stage and a torsional spring (stiffness equal to 100 N∙m/rad), ensuring compliant interaction with the lower-limb segment. The torsional spring deformation is measured by a 17-bit absolute encoder. The second axis (anteriorly located) is collocated with the hip flexion–extension axis and is featured with a 17-bit absolute encoder for hip angle measurement. The two parallel axes are connected by a 4-bar linkage mechanism. For each side, the transfer of assistive torque from the actuation unit to the hip articulation is provided by a thigh cuff connected to the actuation output axis through a rigid link. Each actuation unit can deliver a peak torque of 22 N·m over a range of movement between − 15 and 100 deg and is featured with a passive degree of freedom allowing the user to freely perform hip ab/adduction movements between − 15 and 20 deg.

The APO can deliver the desired torque pattern through a hierarchical control algorithm relying on accurate gait phase recognition for synchronization of the assistive action with the intended movement of the user. The APO control system runs on a real-time controller (NI SbRIO9651 processor, National Instruments, Austin, Texas, US) featured with both a dual-core ARM controller and a Field-Programmable Gate Array (FPGA) processor. A proportional-derivative closed-loop torque compensator running on the FPGA at 1 kHz is responsible for tracking the desired assistive torque (i.e. reference torque) to be delivered to the user. A high-level control layer (running at 100 Hz on the ARM processor) calculates the desired torque profile following a precise gait-phase estimation. The gait phase is estimated by continuously tracking the hip joint angles through a pool of adaptive oscillators and using the maximum hip flexion events for cyclic smooth reset of the gait phase, in accordance with the methodology proposed in^53^.

**Figure 1:**
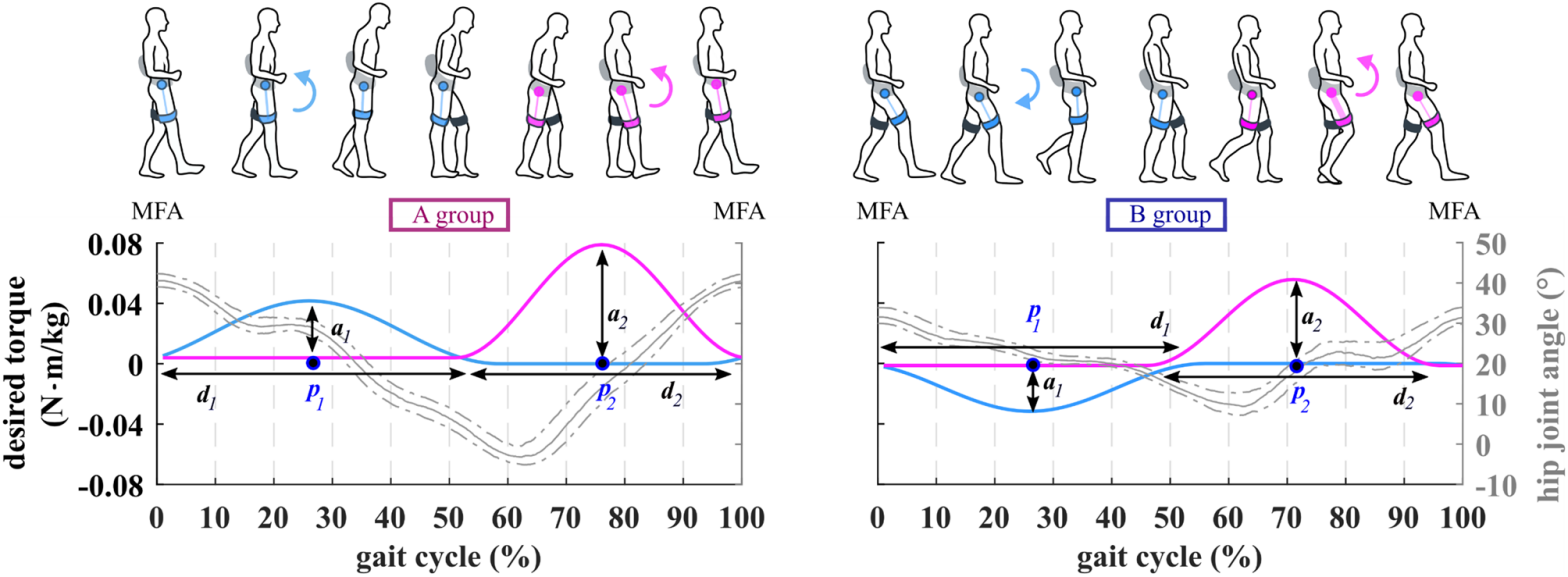
Impairment-specific hip assistance (iHA) adopted for the two study groups. On the left, a double flexion assistive profile adopted for group A (i.e. subjects with knee hyperextension). On the right, an extension-flexion assistive profile adopted for group B (i.e. subjects with deficits in hip flexion/extension). Paretic hip angle (grey), measured by the APO onboard sensors (medians and percentiles—25th, 75th), and desired assistive torque profiles for two representative subjects are depicted. The hip angle is segmented at the max flexion angle (MFA) and normalized to stride duration. The desired assistive torque is computed as the sum of two Gaussian functions (light blue, light purple). The first Gaussian curve occurs during stance, the second during swing. For each Gaussian function, the following parameters can be tuned: phase (p_1_ and p_2_, i.e. % of the gait phase where the peak occurs), amplitude [a_1_ and a_2_ (N·m), i.e. amplitude of the Gaussian peak, which can be either positive (flexion) or negative (extension)] and duration of the assistance (d_1_ and d_2_, i.e. the width of the Gaussian function in % of the gait phase).

The relatively low impedance of the APO combined with the reliable real-time knowledge of the gait phase allows controlling the APO in two different operational modes, namely *transparent mode* (TM) and *assistive mode* (AM). In TM, the controller sets the desired torque to 0 N·m and the APO is *transparent* to the user i.e. the user can walk, and the APO provides minimal-to-null resistance to the user. In AM, the APO provides the phase-locked desired torque. The desired torque is computed for each hip joint, as the sum of two Gaussian functions (each Gaussian function can be used to assist gait either during hip flexion or hip extension)^51^. For each function, the experimenter can tune the following parameters: phase (% of the gait phase where the peak occurs), amplitude (N·m) of the torque peak (positive/negative to deliver flexion/extension assistance respectively) and duration of the assistance (% of the gait phase). The duration is the width of the Gaussian function (Fig. 1).

### Impairment-specific assistive strategy

For the two study groups, different assistive torque profiles were developed for the paretic side (Fig. 1). In particular:

(i) for group A, the assistive torque consisted of a double flexion assistive profile, i.e. an assistive profile with two positive Gaussian curves, the first during the stance phase (synchronously with the knee hyperextension), and the second during the swing phase (we assume that a flexion torque has a positive sign);
(ii) for group B, the assistive torque consisted of an extension-flexion assistive profile, i.e. an assistive profile mimicking the biological hip torque, with a negative Gaussian curve during the stance phase and a positive Gaussian curve during the swing phase.

The design of these assistive profiles underpins the following working hypotheses: (i) for both groups A and B, a flexion torque across the toe-off and during the swing phase can enhance leg propulsion and knee flexion, (ii) for group A, a flexion torque during the stance phase provides resistance to the hip extension which in turn can mitigate knee hyperextension, because of the multi-joint knee-hip dynamics, and (iii) for group B, an extension torque during the stance phase can have a twofold action both supporting the hip extension and stabilizing the knee, e.g. controlling knee buckling.

On the non-paretic side, for both groups A and B, the assistive profile mimicked the biological hip flexion–extension torque: the non-paretic limb can therefore benefit from the assistance and reduce necessary effort from the lack of propulsion of the paretic side.

### Study design

This study lasted 10 months and was a multi-centre interventional longitudinal cohort trial with a single-group design. The study was approved by the local Ethical Committees of the two participating centres, Villa Beretta (VB, Lecco, Italy), and IRCCS Fondazione Don Carlo Gnocchi (FDG, Florence, Italy), and notified to the Italian Ministry of Health. Testing was carried out in accordance with the Declaration of Helsinki (Protocol ID: “Ortesi Pelvica Attiva 01”; approval numbers: 174 for VB and 12738_spe for FDG).

**Figure 2:**
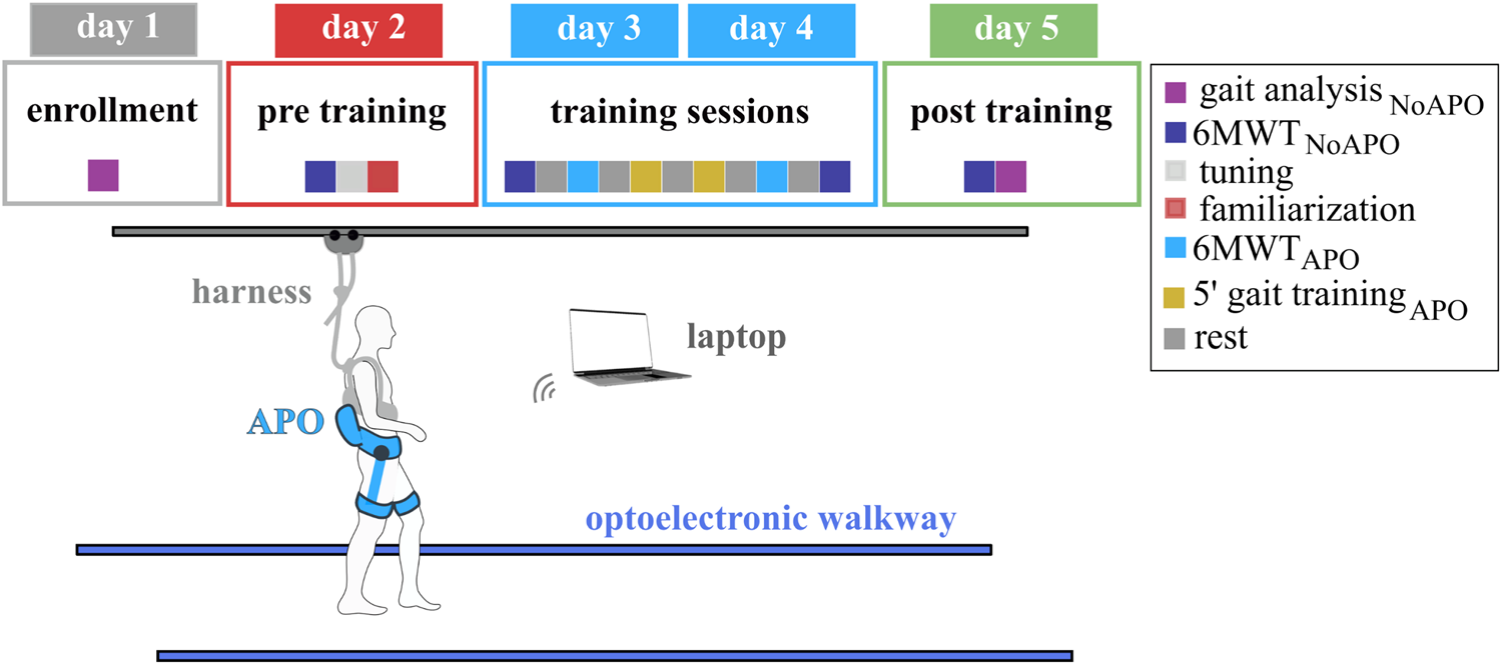
Experimental protocol and setup. The experimental protocol included 5 sessions, namely enrollment, pre-training, training (2x), and post-training sessions. In each session different gait tests and training were executed.

For each participant, the protocol included five sessions conducted on consecutive days (Fig. 2).

In the first session (*Enrolment*), the experimenters determined whether the inclusion and exclusion criteria were met. If the participant was successfully enrolled in the study, the session continued with the participant’s gait analysis (GA): the subject was instructed to walk overground at least 10 times over force platforms along a 10-m walkway in a gait analysis laboratory instrumented with an optoelectronic passive motion capture system while both gait kinematics and kinetics were recorded. Based on the GA records, the clinicians identified the patient’s most relevant gait impairments and assigned the participant to either group A or B.

The second session (*Pre-Training*) had two objectives: to assess walking distance and speed using a 6 Minute Walking Test (6MWT)^54^ and to tune the APO assistive profile. To tune the assistance, the experimenters selected a double-Gaussian torque profile based on group assignment (A or B) and used the kinematic and kinetic profiles from the GA to initialize timing and duration of the torque, i.e. the phases (p_1_ & p_2_) and widths (d_1_ & d_2_) of the profile (Fig. 1). Then, after fitting the subject with the APO, the subject was asked to walk multiple times along an 18-m corridor while the torque assistance (Gaussian amplitudes, a_1_ & a_2_) was gradually increased by the experimenter to let the subject familiarize with the robot’s assistive action. Experimenters fine-tuned the assistive parameters, while the participant walked with the APO, based on visual inspection of the participant’s gait and the real-time plot of the estimated hip joint angle and power. During tuning, the torque assistance was increased until desirable changes in gait were achieved according to clinicians’ feedback, or patient discomfort, or unstable human–robot interaction was noticed.

After setting the final assistive profile the participant was requested to walk for about 10 min at his/her self-selected walking speed to familiarize with the APO assistance.

The third and fourth sessions (*Training*) consisted of a sequence of tests and training trials. At the beginning of the session, the 6MWT was executed twice, i.e. without wearing the APO (6MWT_NoAPO_), and with the APO (6MWT_APO_). Then the subject walked with the APO in AM for 10 min (*gait training*_*APO*_), divided into two walking trials of 5 min each. Lastly, the 6MWT was repeated twice, with and without the APO, i.e. 6MWT_APO_ and 6MWT_NoAPO_. This protocol was conceived to allow the subject to familiarize and train with and without the APO.

During the fifth session (*Post training*), the participant’s gait in the NoAPO condition was assessed with a GA and an additional 6MWT (6MWT_NoAPO_).

Throughout the study, the experimenters noted any device/protocol-related adverse events, device malfunctions (hardware or software) and/or discomfort reported by participants down in the trial diary.

### Outcomes and data analysis

Primary outcomes were the comparison of pre- vs. post-training walking distance and average steady-state speed in the 6MWT (with and without the APO). Secondary outcomes were pre- vs. post-training self-selected gait speed, joint kinematics and kinetics, gait symmetry and forward propulsion, assessed without the APO through 3D gait analysis.

Safety and reliability of the gait training with iHA were assessed as the frequency of device and/or protocol-related adverse events and device malfunctions.

The 6MWTs were conducted on an 18-m corridor. During the 6MWTs, steady-state speed and gait spatiotemporal parameters (e.g. stance time, stride time, and step length) were measured in the middle portion of the corridor using an optoelectronic walkway (Optogait, Microgate, Bolzano, Italy). The perceived effort during each 6MWT was assessed through the CR-10 Borg scale^55^ administered at the end of the test. The heart rate (HR) of the participant was monitored before and after each 6MWT for safety purposes and to allow rest and recovery during consecutive trials.

The GA tests were performed using a stereophotogrammetric motion capture system (Smart-DX, BTS Bioengineering, Quincy, MA, USA) and force platforms (P6000, BTS Bioengineering, Italy). Twenty-two reflective optical markers were placed on anatomical landmarks, following the Davis protocol^56^. Trajectories of optical markets were collected and processed using the BTS Smart software (Smart-DX, BTS Bioengineering, Quincy, MA, USA). Mean and standard errors of the lower-limb kinematic and kinetic profiles were computed. Knee kinematics of the paretic limb was assessed through (i) knee extension peak during the stance phase, (ii) knee flexion peak during the swing phase, and (iii) the gait variable score (GVS) of knee flexion/extension angle profile, computed as the root mean square difference between participant’s and physiological angular profiles^57^. For the hip, knee and ankle of paretic and non-paretic limbs, the range of motion (ROM) was computed as the difference between the maximum and minimum values of the average kinematic profile and the flexion or extension peak torque was computed as the maximum or minimum value of the average kinetic profile.

Finally, data from gait analysis were used to compute self-selected speed, temporal and spatial symmetry, forward propulsion (of the paretic and nonparetic limb), paretic ankle power peak and interlimb propulsion symmetry. The forward propulsion was computed as the mass-normalized peaks [% of body weight (%bw)] of the ground reaction force along the anterior–posterior direction^28,58,59–61^. The spatial, temporal and propulsion symmetry was computed as symmetry ratio ($$SR)$$^62^, as follows:

1 $$SR=\frac{{V}_{paretic}}{{V}_{nonparetic}},$$

where the variable $V$ can be either the step length (cm, in the case of gait spatial symmetry), the stance duration (% gait cycle, in the case of gait temporal symmetry), or the forward propulsion (% bw, in the case of gait propulsion symmetry)^28,63^. Perfect symmetry is given by $SR=1$, whereas the magnitude of asymmetry is defined by ${SR}_{error}=|1-SR|$.

Differences in the distance (in the 6MWT) and the self-selected speed (measured through GA) were compared to the minimal clinically important difference (MCID) scores of 34.4 m^64^ and 0.10 m/s^65^, respectively.

All collected data were analysed by means of custom Matlab routines (MathWorks, Inc., Natick, MA, USA).

### Statistical analysis

The normality of the data distribution was checked by the Kolmogorov–Smirnov statistical inference test (α = 0.05). For all the above-mentioned gait parameters, the statistical test failed to reject the null hypothesis, thus group results have been presented as means and standard errors. To infer statistical differences among sessions, an inferential statistical test was conducted (α = 0.05). For gait analysis outcomes, paired t-tests were used to compare data from pre- and post-training sessions. For 6MWT outcomes, after observing significant differences among sessions through one-way repeated-measures ANOVA, a post-hoc analysis was performed using the Tukey’s honestly significant difference correction. When the sphericity assumption did not hold using the Mauchly’s test, the Epsilon (ꜫ) correction was used [Greenhouse–Geisser correction (ꜫ > 0.75) or Huynh–Feldt correction (ꜫ <  = 0.75), depending on the ꜫ value].

## Results

The clinical protocol was safely completed by all study participants: the APO was used in 42 sessions with non-continuous use of about 25 min per session. During the overall working time of 17 h, no device-related failure, discomfort and/or adverse event was recorded.

### Assistive profiles

The torque amplitudes resulting from the tuning process varied among participants. On the paretic side, during the swing phase, the peak of the flexion torque as measured by the APO ranged between 0.04 and 0.08 N·m/kg. During the stance phase, the peak of the torque ranged between 0.02 and 0.04 N·m/kg (flexion) for group A, and between 0.02 and 0.06 N·m/kg (extension) for group B.

On the non-paretic side, the peak of the flexion torque was in the range [0.04, 0.1] N·m/kg and the peak of the extension torque was in the range [0.02, 0.06] N·m/kg. Assistive profiles of all study participants are reported in the supplementary materials (Supplementary Fig. 1).

### Primary outcomes: walking distance and speed in 6MWTs

**Figure 3:**
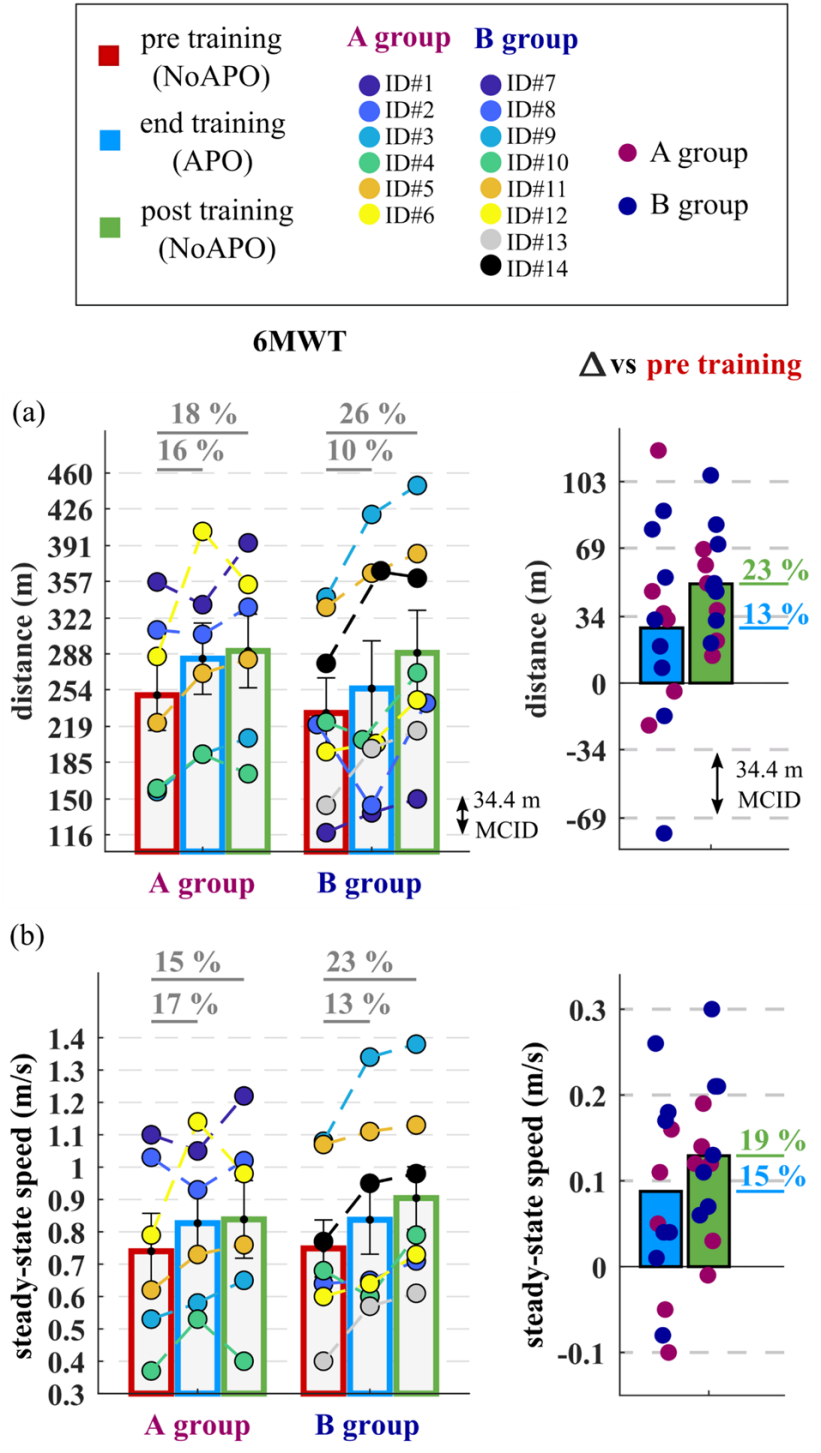
Results of the 6MWTs pre and post-training. (**a**) Walked distance and (**b**) steady-state speed during 6MWTs. Data are reported for each study group in bar plots as means and standard errors (left) and as differences between pre-training (NoAPO) and training (APO) and post-training (NoAPO) (right). The minimal clinically important difference (MCID) for the distance is depicted in each plot with dashed horizontal lines. Circles represent individual study participants.

Results of the walked distance and gait speed achieved in the 6MWTs at the main timepoints [i.e. pre- and post-training without APO (6MWT_NoAPO_) and at the end of the training with APO (6MWT_APO_)] are reported in Fig. 3.

After 2 days of training, study participants significantly increased the walked distance on average by 23 ± 3% (51 ± 7 m, p < 0.0001). Both groups A and B showed a statistically (p < 0.05) and clinically (> 34.4 m MCID^64^) significant increment of the walked distance, respectively by 42 ± 9 m (p = 0.01) and 57 ± 10 m (p = 0.002), comparing pre-training NoAPO (249 ± 34 m group A, 232 ± 33 m group B) with post-training NoAPO (291 ± 35 m group A, 289 ± 41 m group B). When walking with the APO, the walked distance increased on average by 13 ± 6% (28 ± 13 m, p = 0.1) comparing the 6MWT_APO_ at the end of the training (283 ± 34 m group A, 255 ± 45 m group B) with the pre-training 6MWT_NoAPO_. With the APO, most participants approached or surpassed the MCID, while one subject of group B (ID#8), reduced the walked distance (by 77 m) due to intermittent claudication. Excluding the data from the subject that stopped during the test (ID#8), both groups A and B showed a clinically (> MCID) significant increment of the walked distance, respectively, by 35 ± 20 m and 39 ± 14 m [Fig. 3a].

After the training, participants significantly increased the steady-state speed during the 6MWT on average by 19 ± 4% (0.13 ± 0.02 m/s, p < 0.001). Both groups A and B increased their steady-state speed, respectively by 0.10 m/s (p = 0.05) and 0.16 m/s (p = 0.008), from pre-training NoAPO (0.74 ± 0.29 m/s group A, 0.75 ± 0.25 m/s group B) to post-training NoAPO (0.84 ± 0.29 m/s group A, 0.90 ± 0.27 m/s group B). When walking with the APO, participants increased their steady-state speed on average by 15 ± 5% (0.09 ± 0.04 m/s, p = 0.03) comparing the 6MWT_APO_ at the end of the training (0.83 ± 0.25 m/s group A, 0.84 ± 0.30 m/s group B) with the pre-training 6MWT_NoAPO_ [Fig. 3b].

The self-assessment of the task intensity measured using the Borg Scale did not change significantly from pre- to post-training for both groups. Borg scale scores, heart rate and walked distance of all 6MWTs performed are reported in supplementary materials (Supplementary Fig. 2, Supplementary Table III).

### Secondary outcomes

#### Self-selected gait speed

**Figure 4:**
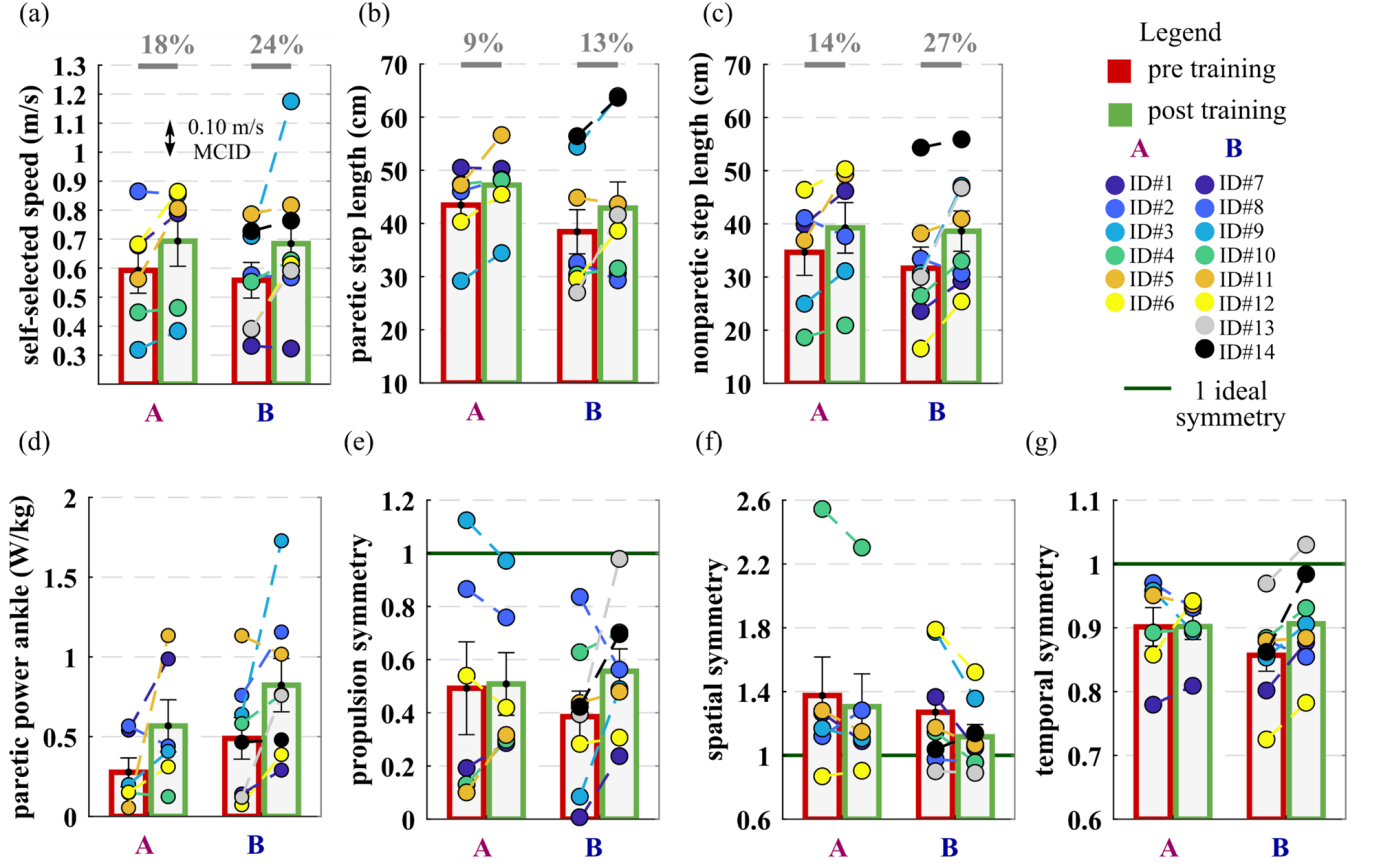
Self-selected speed, spatiotemporal and propulsion parameters pre and post-training. (**a**) Self-selected gait speed (m/s), (**b**) paretic and (**c**) non-paretic step length (cm), (**d**) paretic power ankle (W/kg), (**e**) propulsion symmetry, (**f**) spatial and (**g**) temporal gait symmetry measured during pre (red) and post (green) training gait analysis are shown averaged across group participants (means and standard errors). Circles represent individual study participants. The minimal clinically important difference (MCID) for the speed is depicted in the plot with dashed horizontal lines.

GA data showed a significant increase in the self-selected walking speed of 21 ± 6% (0.12 ± 0.04 m/s, p = 0.007) after 2-day of training [Fig. 4a]. In particular, the self-selected walking speed increased on average by 18 ± 6% in group A and by 24 ± 10% in group B, from pre-training (0.59 ± 0.08 m/s group A, 0.56 ± 0.06 m/s group B), to post-training (0.69 ± 0.09 m/s group A, 0.68 ± 0.09 m/s for group B). On average, both groups showed a clinically meaningful change (≥ 0.10 m/s MCID^65^) in the comfortable walking speed, respectively by 0.10 m/s for group A and 0.13 m/s for group B.

#### Gait symmetry

On average, study participants significantly improved the spatial symmetry by 0.11 ± 0.04 (p = 0.03), when comparing pre-training (1.32 ± 0.12) and post-training (1.20 ± 0.08). The spatial gait symmetry improved respectively by 0.08 ± 0.06 for group A and by 0.14 ± 0.06 for group B [Fig. 4f].

This improvement in the spatial symmetry resulted from a significant increase in step length on both the paretic and non-paretic limbs, by 11 ± 4% (4 ± 1 cm, p = 0.01) and by 21 ± 6% (6 ± 2 cm, p = 0.003) respectively [Fig. 4b,c].

The temporal symmetry significantly improved in group B by 0.04 ± 0.02 (p = 0.03), when comparing pre-training (0.86 ± 0.03) and post-training (0.91 ± 0.03) GAs, while in group A, on average, it did not change (0.00 ± 0.02), [Fig. 4g].

#### Forward propulsion and propulsion symmetry

After 2 days of gait training, study participants significantly increased their paretic ankle power, on average by 0.32 W/kg (p = 0.009). Specifically, the paretic ankle power increased by 0.29 ± 0.11 W/kg in group A and 0.33 ± 0.10 W/kg in group B comparing pre (0.28 ± 0.09 W/kg group A, 0.49 ± 0.13 W/kg group B) and post-training GA [Fig. 4d].

On average, study participants had a non-significant increase in the paretic limb forward propulsion by 1.10 ± 0.66%bw (p = 0.1) when comparing pre-training (3.17 ± 0.71%bw) and post-training (4.27 ± 0.68%bw) GAs. These average changes contributed to considerable improvement in the interlimb propulsion symmetry of 0.12 ± 0.06 (p = 0.1); in particular of 0.06 ± 0.06 in group A and 0.17 ± 0.09 in group B.(p > 0.05), see Fig. 4e.

#### Joint kinematics

**Figure 5:**
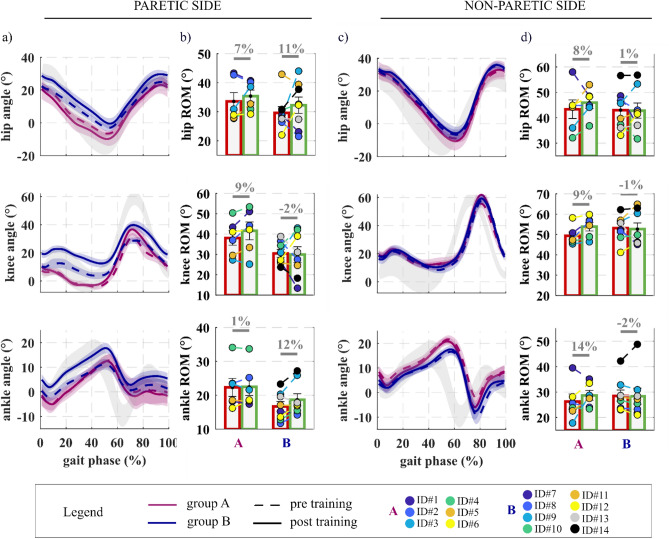
Lower-limb kinematics averaged across group participants pre and post training. Joint kinematic profiles and range of motion (ROM) of the paretic (**a,b**) and non-paretic (**c,d**) hip, knee and ankle joints measured during the pre-training (dashed lines) and post-training (solid lines) gait analysis for the group A (purple) and the group B (blue). Data are shown aggregated across group participants as mean ± standard errors (n = 6 group A, n = 8 group B). Circles represent individual study participants. Normative data for the hip, knee and ankle kinematic profiles are reported in light grey.

Hip, knee and ankle kinematic profiles and ROM of the paretic and non-paretic limbs are reported in Fig. 5. There were no statistically significant differences in joints ROM between pre and post-training. The ROM of the paretic hip joint was slightly larger for both groups A (7%, p = 0.3) and B (11%, p = 0.3) post-training than pre-training.

**Figure 6:**
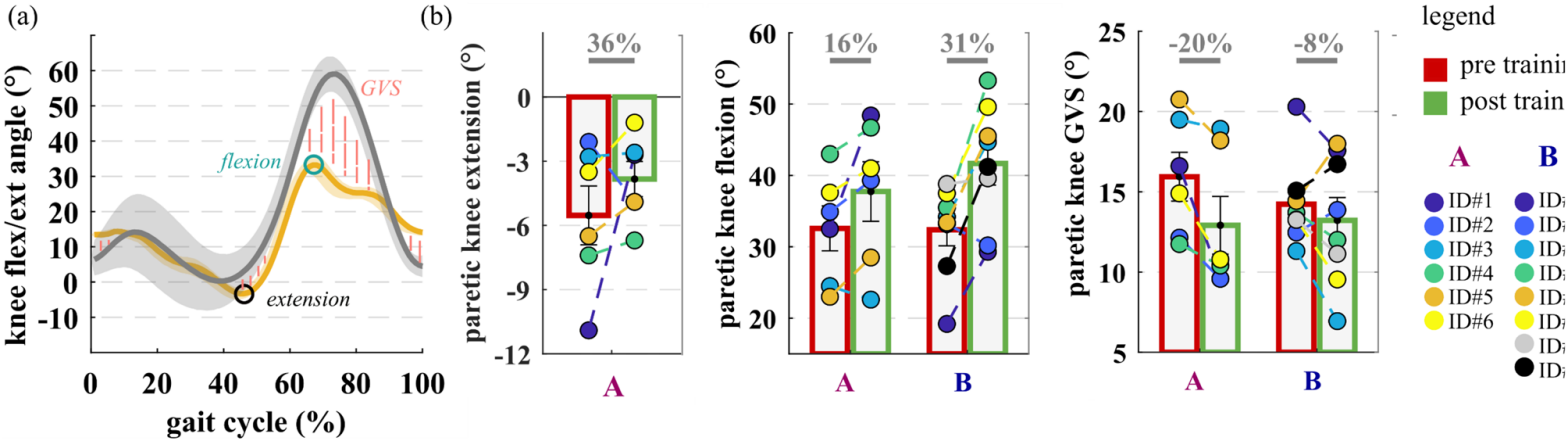
Paretic knee kinematics pre and post training. (**a**) A graphical schematic representation of parameters considered for the analysis. Knee flexion–extension profiles of healthy subjects (grey) and of a study participant (orange) are depicted. (**b**) From left to right, paretic knee extension peak during stance for the A group, paretic knee flexion peak during swing and gait variable score (GVS) for the paretic knee flexion/extension angle profile for both groups A and B, measured during gait analysis pre (red) and post (green) training are shown. Grouped data (i.e. group A, group B) are reported in bar plots as mean and standard errors. Circles represent individual study participants.

In group A, paretic-limb knee hyperextension (knee extension peak) was reduced by 2 degrees (36%) on average when comparing post-training (− 4 ± 1°) with pre-training (− 6 ± 1°) in the NoAPO condition. Although five out of six subjects decreased the intensity of knee hyperextension when comparing results from GA at post-training with the pre-training, this change was not statistically significant (p = 0.3). The peak knee flexion during swing significantly increased by 24 ± 6% (p = 0.0009) post-training (40 ± 2°) compared to pre-training (32 ± 2°), specifically by 16% (5° ± 2°, p = 0.08) and 31% (9° ± 2°, p = 0.007), respectively for the A and B groups (Fig. 6). However, while in group A, this increment was concomitant to an increase of the knee ROM (by 9 ± 4%, p = 0.07), in the group B it was due to a more flexed knee pattern profile throughout the whole gait cycle (corresponding to a not significant change in knee ROM of − 2 ± 11%, p = 0.8 pre- vs post-training). Nevertheless, the GVS for the knee flexion/extension angle for both groups A and B significantly decreased (indicating a higher similarity with the normative profile) post-training compared to pre-training (2 ± 1°, p = 0.02). In particular, the change was significant by 20 ± 6% (3 ± 1°, p = 0.02) in group A and not significantly by 8 ± 7% (1 ± 1°, p = 0.3) in group B.

On the non-paretic side, the hip, knee and ankle ROM increased only for group A [8% (p = 0.4), 9% (p = 0.02) and 14% (p = 0.3) respectively], while on the paretic side the ankle ROM increased only for the B group (12%, p = 0.06).

#### Joint kinetics

**Figure 7:**
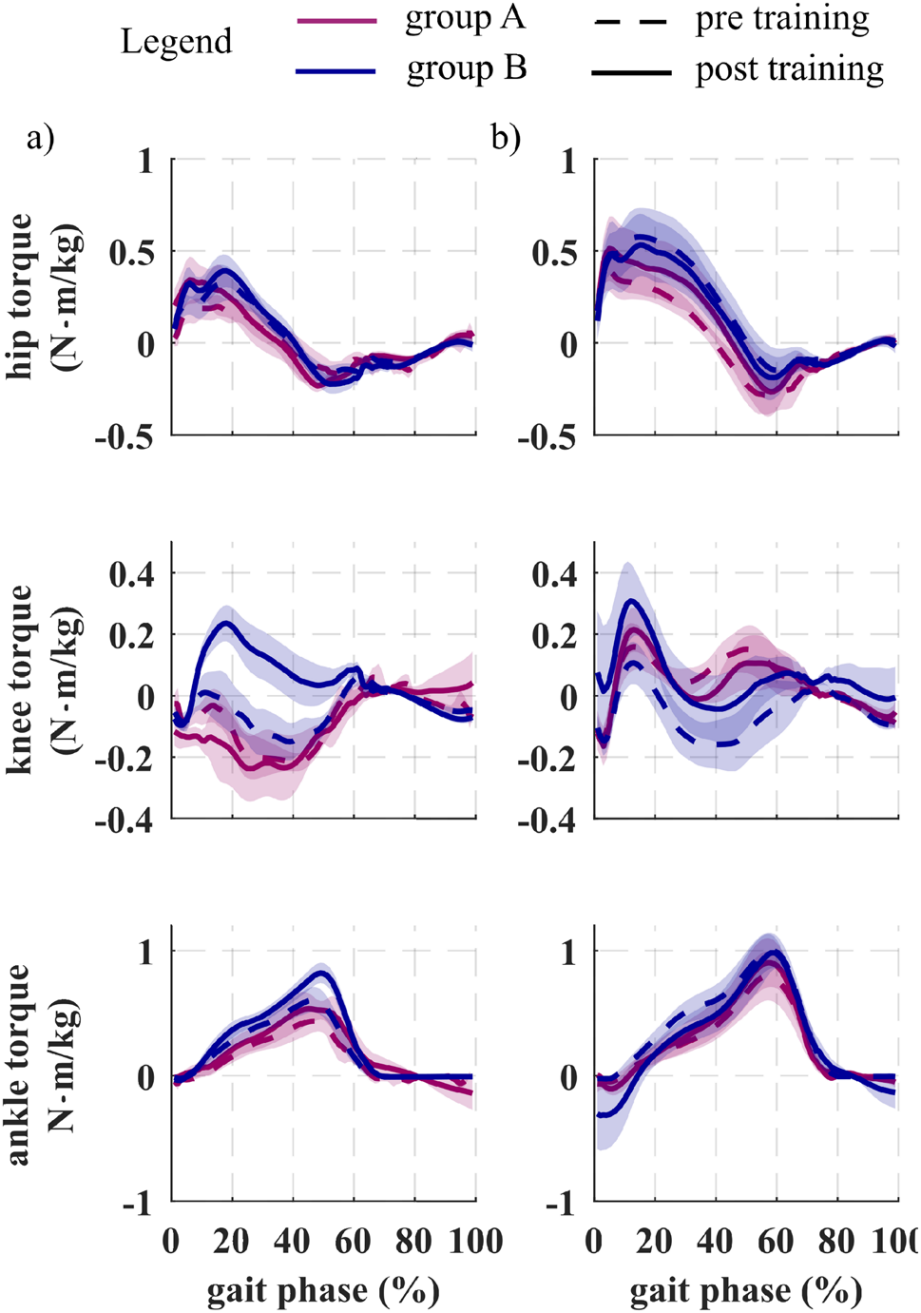
Lower-limb kinetics averaged across group participants pre and post training. Joint kinetic profiles of the paretic (**a**) and non-paretic (**b**) hip, knee and ankle joints measured during the pre-training (dashed lines) and post-training (solid lines) gait analysis for the group A (purple) and the group B (blue). Data are shown aggregated across group participants as mean ± standard errors (n = 6 group A, n = 8 group B).

Hip, knee and ankle flexor/extensor torque profiles are reported in Fig. 7. Following the training, study participants increased their mean hip extension torque both on the paretic and non-paretic side, respectively by 0.11 ± 0.05 N∙m/kg, p = 0.07, (0.12 ± 0.06 N∙m/kg A, 0.09 ± 0.05 N∙m/kg B) and 0.07 ± 0.06 N∙m/kg, p = 0.3 (0.11 ± 0.08 N∙m/kg A, 0.04 ± 0.05 N∙m/kg B). The knee extension torque increased only in group B both on the paretic and non-paretic side, respectively by 0.16 ± 0.05 N∙m/kg, p = 0.5 and 0.28 ± 0.14 N∙m/kg, p = 0.2. This was not the case in group A (0.04 ± 0.06 N∙m/kg paretic and 0.04 ± 0.04 N∙m/kg non-paretic side). There was no relevant change in hip flexion torque for the paretic and non-paretic limbs when comparing post vs. pre-training. Post-training both groups significantly increased their plantarflexion torque on the paretic limb, on average by 0.20 ± 0.08 N∙m/kg (p = 0.03), without relevant change on the non-paretic limb (0.05 ± 0.05 N∙m/kg).

## Discussion

This pilot study investigated (i) the feasibility of providing the proposed iHA using an APO for patients with mild-to-moderate neurological gait impairments related to ABI, (ii) the potential effects of a short robot-mediated training with iHA, primarily, on functional outcomes, as walking distance and speed during 6MWTs and secondarily, on gait kinematics and kinetics.

Throughout the 17 h of use, the APO delivered safe and reliable hip assistance to all study participants. No adverse events, malfunctioning and/or discomfort were reported during the use of the device and all subjects successfully completed the assigned study tasks. This result traces a positive perspective to design larger studies where the APO can be used for even longer and more intensively, where device safety is purposively and systematically assessed, with methodologies similar to the one reported in^66^. Clinicians selected the assistive profile using clinical judgement based on instrumented evaluation of the patients using gait analysis. For the proposed methodology, gait evaluation and the involvement of experienced clinical staff to configure and fine-tune the APO assistance are believed to be critical to achieving effective human–robot interaction and beneficial effects on gait.

In this study, we focused primarily on the effectiveness of iHA on functional outcomes, walking endurance based on the 6MWT and speed, as they are clinically-salient measures that have a strong association with community engagement and provide a common measure for the effectiveness of rehabilitation^46,47^. The short APO-mediated training with iHA led to a statistically and clinically significant increase in distance and speed in the 6MWTs, both with and without the APO (6MWT_APO_ and 6MWT_NoAPO_). After the two APO-mediated training sessions, the walked distance during the 6MWT increased by 51 m (about 23%) on average: this result is comparable to other studies in the literature, which reports an increase of (i) 40 m after 18 sessions of task-oriented training to enhance walking distance and speed^49^, or (ii) 53 m after 36 sessions of a high-intensity aerobic treadmill training in elderly chronic individuals^67^, or (iii) 59 m after 24 sessions of a home-based exercise program in individuals with mild and moderate stroke^68^. Other studies reported increments from 11 to 97 m after respectively 12 and 48 sessions of training as reported in a review on RAGT for post-stroke patients ^12^.

Given the shorter duration and the uncontrolled design of our study, a fair discussion of the achieved results shall consider that the observed increment of distance and speed may be also explained by two concomitant (confounding) effects: (i) practising with the 6MWT, and (ii) weight training. Indeed, in addition to the short-term retention of an adapted motor control strategy, as a consequence of the energy injected by the APO into the gait scheme, individuals may have, on the one hand, gained proficiency in performing the 6MWT over the eight iterations, and, on the other hand, be exposed to a weight training, given the not-neglectable weight of APO.

As it regards the practising effect, some studies have challenged the reliability of the 6MWT as an assessment test with respect to the possible practice effect. While some studies point out that the practice effect can lead to an increase in the walked distance (for a 2–8% learning effect between repeated 6MWTs)^69^ other studies have concluded that the 6MWT has a clear and strong ability to demonstrate change in clinical status, given its high test–retest reliability when used to assess individuals with stroke^70^.

For what concerns the weight training the most representative study in the literature is^71^ In their randomized control trial, Shi and Lee have pointed out that post-stroke patients who train their walking ability while wearing an additional weight (7% of the body mass) can increase significantly their speed up to about 100%. This result is achieved after a much longer training program (18 sessions) compared to our study and does not offer any indication to estimate the impact of weight training in shorter training protocols.

From the above analysis, it is not possible to quantitatively estimate how much the practice effect and weight training have had an impact on the observed increment of distance and speed. However, we are also quite confident in stating that the training period in our study is sufficiently short to exclude that the observed increase in distance and speed can be motivated by the sole action of the practice and weight training effects. This consideration is also partly supported by the fact that we observed also qualitative gait changes in terms of gait kinematics, propulsion and symmetry, which are an additional indication of the fact that the APO action had an impact on the body control scheme of the participants.

Being capable of stating that the APO may have had the capability to constructively interfere with the body control scheme of the recruited post-stroke individuals is sufficient to consider this study as a positive feasibility test, preparatory for a more structured randomized controlled trial. Likely, a randomized control study will allow us to isolate the effect of the iHA-based gait training from any confounding effect and -thanks to a longer training program- elucidate whether this motor learning process could lead to a more efficient, effective and longer-term motor/functional recovery.

Giving a closer look at the results of 6MWTs, there is a discrepancy between the variation of distance and steady-state speed with and without the APO. Compared to pre-training, in the 6MWT_APO_ the improvement in steady-state speed (15%) was higher than in distance (13%), while in the 6MWT_NoAPO_, the improvement in the distance (23%) was higher than in steady-state speed (19%). This difference can be explained by the inherent nature of the 6MWT which is composed of steady-state (mid part of the corridor) and transient (deceleration, turning, and acceleration at the end of each corridor) phases. While the patients seemed to benefit from the assistance during the steady-state phase when walking with the APO (increased steady-state walking speed), that might have been hindered by the APO during the transient phases, due to the need to accelerate and decelerate the additional mass of the APO. Conversely, in the 6MWT_NoAPO_, the higher increment in distance than in steady-state speed might be explained by the familiarization process with the task, especially with faster execution of the turns.

In addition to the increase in walking speed, measurement of gait quality, including gait kinematics and kinetics, should also be considered to better characterise neuromotor recovery^72^ In fact, compensatory intra- and inter-limb movements could lead to higher walking speed, despite persisting impairments and unstable gait. Hence, it is worth noting that in this study, improvements in self-selected comfortable walking speed (on average, 0.12 m/s) were observed in parallel with small improvements in joint kinematics, gait symmetry and propulsion symmetry, therefore, suggesting an overall improvement and more efficient gait.

Improvements in spatial symmetry were in line with those reported in a recent review on RAGT for post-stroke patients^73^, which were in the range [0.01–0.6] for the robot group, and [0.04–0.4] for the control group.

Similarly, improvements in propulsion symmetry reported in this study were comparable with those published in other studies using exoskeletons that directly assist ankle plantarflexion^28^. The presented approach, also, confirms the results of previous studies on healthy subjects that suggested the influence of hip training on gait propulsion^74^.

Although the statistical comparison of the two groups was not an objective of this pilot study, we noted a slight difference between groups A and B in results in terms of spatial and temporal symmetry as well as in paretic limb forward propulsion and propulsion symmetry. Overall, group B showed higher improvements in all the symmetry indicators, suggesting that the APO hip extension torque provided for the paretic limb during the stance phase may be associated with greater propulsion^72^ which, in turn, can promote longer steps and higher duration of the stance phase. This result may denote that the iHA, conceived to mitigate the knee hyperextension (adopted for group A), might require longer training for promoting also the development of a symmetric gait scheme. In addition, this difference between the study groups in terms of symmetry was concurrent with a different kinematic change in terms of ROM of the hip and knee joints. In fact, in group B the hip and knee ROM increased on the paretic limb without parallel changes on the nonparetic limb, while in group A the joint ROMs increased both on the paretic and nonparetic limb. This might suggest that the customization of the hip robotic assistance could be needed also for the non-paretic limb to achieve more global improvements in gait.

For the first time, we proposed and tested the use of hip flexion torque during stance to reduce knee hyperextension. Five out of six patients, who presented with knee hyperextension, showed a decreased hyperextension angle and also an increased hip extension angle, following the 2 days of training with the hip flexion torque during the stance phase. The hip extension angle increased more in the A group (that received resistive hip extension torque during training) than in the B group (that received assistive hip extension torque). This result might suggest that the novel hip flexion torque profile proposed to mitigate knee-hyperextension during stance could also be used to strengthen hip extensors through resistive training.

We also explored the use of hip flexion assistance to address the deficit in knee flexion during swing (common post-ABI gait deviation). Twelve out of fourteen patients showed an increase in knee flexion angle in the swing after training.

During normal walking, swing knee flexion is generated primarily by the concentric contraction of hip flexors, as the leg in swing act as a compound pendulum^75,76^. In preparation for swing, the knee is accelerated into flexion during the double support phase by reaching the flexion velocity peak at the toe-off. The magnitude of the velocity peak in the pre-swing phase is crucial to achieving a sufficient knee flexion late in the swing phase^77^. Among contributors to knee flexion velocity in pre-swing are: (i) an external knee flexion moment, (ii) ankle plantarflexion at push-off phase and (iii) hip flexors (mainly iliacus and psoas activation) at the pull-off phase, and all three factors can be impaired by stroke. Thus, by enhancing hip flexion at the pull-off phase (e.g. through the assistive action of the APO) the knee flexion could increase during the swing phase.

From a mechanical point of view, during walking, the lower-limb segments and the pelvis alternatively form an open and closed kinetic (kinematic) chain depending on the phase (swing and stance). Both in closed or open chain, an external force applied to one segment is transferred to an adjacent segment, enabling a cumulative response of the whole chain. Previous work showed a strong correlation between the hip and the knee during the entire stance phase and the mid swing, in the sagittal plane^45^. The closed kinematic chain is manipulated also by an AFO to change the knee angle during stance^43^.

A previous pilot study, using a hip flexion exosuit with three post-stroke individuals showed a small effect on knee kinematics (one subject increased knee flexion in swing, one subject reduced knee hyperextension). Furthermore, a study testing hip-knee-ankle torque assistance delivered in different single- or multiple-joint combinations showed that the indirect effects of hip-only and ankle-only assistance on the knee during stance were larger than the direct effect of knee assistance^78^ Overall, these studies show that torque assistance at the hip can affect knee kinematics, if confirmed by future investigations, the novel approach proposed in this study (i.e. iHA) can open new alternatives to the implementation of assistive devices to enhance gait and reduce knee-related deviations.

A rigorous comparison of the results of this study with similar studies in the literature is not possible because of several differences in the training programs^73,79^ In particular, the majority of the studies included longer training programs, and in many cases, they included control groups to determine whether the intervention had a significant effect^12,73^. In our study, the short duration of the training and the lack of a control group, do not allow a straightforward interpretation of the results. The longer walked distance observed in the post-training session compared to the pre-training session (on average, 51 m), may have been due to a combination of the training duration and intensity (during the two training days all subjects walked significantly more than they are used to) and we provided additional gait assistance through the APO. The specific effect of the APO assistance provided could be supported by the observed improvements in gait quality (either assessed by global indexes, spatiotemporal symmetry and propulsion and joint-specific indexes, such as knee hyperextension angle), which in turn may be more likely attributable to the short-term effect of a different gait scheme provided through iHA, not related to a simple increase in exercise volume and intensity. While the above considerations may suggest a positive effect of the iHA, future investigations must be focused on isolating the effects of the APO-based assistance, by including control conditions as well as a larger sample trained over a longer time period.

Concerning the knee hyperextension reduction strategy, an accurate diagnosis of the causes that produce the impairment is relevant for the effectiveness of the suggested hip flexion torque and safety during the stance phase. For example, in patients with significant knee muscle weakness, they may develop knee hyperextension as a compensatory strategy to prevent the knee from collapsing, the proposed iHA strategy could be inappropriate or even unsafe in such cases. Thus, further investigation of the proposed iHA strategy should consider these aspects in more depth.

This study focused mainly on two categories of common gait disorders. The repertoire of possible ABI-induced gait disorders includes several more, so future studies may investigate a wider spectrum of personalized assistive profiles. However, the proposed iHA customization strategy, should be considered only as a guideline for clinicians, to be substantiated with the patient in the loop and if necessary, to be modulated throughout treatment.

In this study, the identification of the subject’s most relevant gait impairment as well as the process to identify subject-specific control parameters (e.g. timing and magnitude of the assistance) was based on visual and clinical assessment of the gait profiles by the clinicians and their manual tuning of the parameters. Such a manual tuning may result in sub-optimal control conditions, as previous works showed that small changes in torque profiles can result in more or less pronounced gait changes^74^. Furthermore, a recent trend in the wearable robotics field is the use of “human-in-the-loop tuning optimization”, to find the optimal set of control parameters to maximize a specific outcome; which in most cases, consisted in the metabolic cost of walking^80^. While we recognize that human-in-the-loop tuning optimization processes may not be applicable to ABI rehabilitation, as they require long walking time, which is challenging for individuals with gait impairments and incompatible with the established clinical rehabilitation protocols; future investigations should focus on the development of automated procedures to increase the robustness of our method and help clinicians through the selection of appropriate assistive parameters.

## Supplementary Information

Supplementary Information.

## Data Availability

The datasets generated and/or analysed during the current study are available from the corresponding author on reasonable request. The methodology proposed in this manuscript is covered by the patent WO2022137031 “System and method for correcting gait impairments”, June 30th, 2022.

## References

[1] Virani SS (2020). Heart disease and stroke statistics—2020 update: A report from the American Heart Association. Circulation.

[2] Perry J, Garrett M, Gronley JK, Mulroy SJ (1995). Classification of walking handicap in the stroke population. Stroke.

[3] Gresham GE (1975). Residual disability in survivors of stroke—The Framingham study. N. Engl. J. Med..

[4] Schmid A (2007). Improvements in speed-based gait classifications are meaningful. Stroke.

[5] Harris JE, Eng JJ (2004). Goal priorities identified through client-centred measurement in individuals with chronic stroke. Physiother. Can..

[6] Bohannon RW, Andrews AW, Smith MB (1988). Rehabilitation goals of patients with hemiplegia. Int. J. Rehabil. Res..

[7] Winstein CJ (2016). Guidelines for adult stroke rehabilitation and recovery: A guideline for healthcare professionals from the American Heart Association/American Stroke Association. Stroke.

[8] Calabrò RS (2016). Robotic gait rehabilitation and substitution devices in neurological disorders: Where are we now?. Neurol. Sci..

[9] Morone G (2017). Robot-assisted gait training for stroke patients: Current state of the art and perspectives of robotics. Neuropsychiatr. Dis. Treat..

[10] George Hornby T (2011). Importance of specificity, amount and intensity of locomotor training to improve ambulatory function in patients poststroke. Top. Stroke Rehabil..

[11] Takao T (2015). Improvement of gait ability with a short-term intensive gait rehabilitation program using body weight support treadmill training in community dwelling chronic poststroke survivors. J. Phys. Ther. Sci..

[12] Mehrholz J, Thomas S, Kugler J, Pohl M, Elsner B (2020). Electromechanical-assisted training for walking after stroke. Cochrane database Syst. Rev..

[13] Molteni F, Gasperini G, Cannaviello G, Guanziroli E (2018). Exoskeleton and end-effector robots for upper and lower limbs rehabilitation: narrative review. PMR.

[14] Spiess MR, Steenbrink F, Esquenazi A (2018). Getting the best out of advanced rehabilitation technology for the lower limbs: Minding motor learning principles. PMR.

[15] Esquenazi A, Talaty M, Jayaraman A (2017). Powered exoskeletons for walking assistance in persons with central nervous system injuries: A narrative review. PMR.

[16] He Y, Eguren D, Luu TP, Contreras-Vidal JL (2017). Risk management and regulations for lower limb medical exoskeletons: A review. Med. Devices.

[17] Palermo AE, Maher JL, Baunsgaard CB, Nash MS (2017). Clinician-focused overview of bionic exoskeleton use after spinal cord injury. Top. Spinal Cord Inj. Rehabil..

[18] Hendricks HT, Van Limbeek J, Geurts AC, Zwarts MJ (2002). Motor recovery after stroke: A systematic review of the literature. Arch. Phys. Med. Rehabil..

[19] Veale AJ, Xie SQ (2016). Towards compliant and wearable robotic orthoses: A review of current and emerging actuator technologies. Med. Eng. Phys..

[20] Buesing C (2015). Effects of a wearable exoskeleton stride management assist system (SMA®) on spatiotemporal gait characteristics in individuals after stroke: A randomized controlled trial. J. Neuroeng. Rehabil..

[21] Wright A (2018). A community-based, bionic leg rehabilitation program for patients with chronic stroke: Clinical trial protocol. J. Stroke Cerebrovasc. Dis..

[22] Lee HJ (2019). Training for walking efficiency with a wearable hip-assist robot in patients with stroke a pilot randomized controlled trial. Stroke.

[23] Lynch E, Hillier S, Cadilhac D (2014). When should physical rehabilitation commence after stroke: A systematic review. Int. J. Stroke.

[24] Wang, K. et al. Clinical research of lower extremity exoskeleton robot In post-stroke hemiplegic patients. 1–22 doi:10.21203/rs.3.rs-36484/v1(2020).

[25] Jayaraman A (2019). Stride management assist exoskeleton vs functional gait training in stroke: A randomized trial. Neurology.

[26] Jayaraman A (2019). Immediate adaptations to poststroke walking performance using a wearable robotic exoskeleton. Arch. Phys. Med. Rehabil..

[27] Awad LN (2017). Reducing circumduction and hip hiking during hemiparetic walking through targeted assistance of the paretic limb using a soft robotic exosuit. Am. J. Phys. Med. Rehabil..

[28] Awad LN (2017). A soft robotic exosuit improves walking in patients after stroke. Sci. Transl. Med..

[29] Bae J (2018). Biomechanical mechanisms underlying exosuit-induced improvements in walking economy after stroke. J. Exp. Biol..

[30] Duschau-Wicke A, Caprez A, Riener R (2010). Patient-cooperative control increases active participation of individuals with SCI during robot-aided gait training. J. Neuroeng. Rehabil..

[31] Goffredo M (2019). Overground wearable powered exoskeleton for gait training in subacute stroke subjects: Clinical and gait assessments. Eur. J. Phys. Rehabil. Med..

[32] Olney SJ, Richards C (1996). Hemiparetic gait following stroke part I: Characteristics. Gait Posture.

[33] Woolley SM (2001). Characteristics of gait in hemiplegia. Top. Stroke Rehabil..

[34] Balaban B, Tok F (2014). Gait disturbances in patients with stroke. PMR.

[35] Cooper A, Alghamdi GA, Alghamdi MA, Altowaijri A, Richardson S (2012). The relationship of lower limb muscle strength and knee joint hyperextension during the stance phase of gait in hemiparetic stroke patients. Physiother. Res. Int..

[36] Geerars M, Minnaar-van der Feen N, Huisstede BM (2022). Treatment of knee hyperextension in post-stroke gait. A systematic review. Gait Posture.

[37] Caillet F, Mertens P, Rabaseda S, Boisson D (1998). The development of gait in the hemiplegic patient after selective tibial neurotomy. Neurochirurgie.

[38] Boudarham J (2013). Effects of a knee-ankle-foot orthosis on gait biomechanical characteristics of paretic and non-paretic limbs in hemiplegic patients with genu recurvatum. Clin. Biomech..

[39] Requier B (2018). Knee-ankle-foot orthoses for treating posterior knee pain resulting from genu recurvatum: Efficiency, patients’ tolerance and satisfaction. J. Rehabil. Med..

[40] Portnoy S, Frechtel A, Raveh E, Schwartz I (2015). Prevention of genu recurvatum in poststroke patients using a hinged soft knee orthosis. PMR.

[41] Dalal KK (2018). Effectiveness of prowling with proprioceptive training on knee hyperextension among stroke subjects using videographic observation—A randomised controlled trial. Gait Posture.

[42] Bae D-Y, Shin J-H, Kim J-S (2019). Effects of dorsiflexor functional electrical stimulation compared to an ankle/foot orthosis on stroke-related genu recurvatum gait. J. Phys. Ther. Sci..

[43] Kobayashi T (2016). Reduction of genu recurvatum through adjustment of plantarflexion resistance of an articulated ankle-foot orthosis in individuals post-stroke. Clin. Biomech..

[44] Bleyenheuft C, Bleyenheuft Y, Hanson P, Deltombe T (2010). Treatment of genu recurvatum in hemiparetic adult patients: A systematic literature review. Ann. Phys. Rehabil. Med..

[45] Svoboda Z, Janura M, Kutilek P, Janurova E (2016). Relationships between movements of the lower limb joints and the pelvis in open and closed kinematic chains during a gait cycle. J. Hum. Kinet..

[46] Fulk GD, He Y, Boyne P, Dunning K (2017). Predicting home and community walking activity poststroke. Stroke.

[47] Awad L, Reisman D, Binder-macleod S (2019). Distance-induced changes in walking speed after stroke: Relationship to community walking activity. J. Neurol. Phys. Ther..

[48] Mehrholz J, Wagner K, Rutte K, Meißner D, Pohl M (2007). Predictive validity and responsiveness of the functional ambulation category in hemiparetic patients after stroke. Arch. Phys. Med. Rehabil..

[49] Salbach NM (2004). A task-orientated intervention enhances walking distance and speed in the first year post stroke: A randomized controlled trial. Clin. Rehabil..

[50] Giovacchini F (2015). A light-weight active orthosis for hip movement assistance. Robot. Auton. Syst..

[51] Martini E (2019). Gait training using a robotic hip exoskeleton improves metabolic gait efficiency in the elderly. Sci. Rep..

[52] Pratt GA, Williamson MM (1995). Series elastic actuators. IEEE Int. Conf. Intell. Robot. Syst..

[53] Yan T (2017). An oscillator-based smooth real-time estimate of gait phase for wearable robotics. Auton. Robots.

[54] Kennedy DM, Stratford PW, Wessel J, Gollish JD, Penney D (2005). Assessing stability and change of four performance measures: A longitudinal study evaluating outcome following total hip and knee arthroplasty. BMC Musculoskelet. Disord..

[55] Borg G (1998). Borg’s perceived exertion and pain scales.

[56] Davis RB, Ounpuu S, Tyburski D, Gage JR (1991). A gait analysis data collection and reduction technique. Hum. Mov. Sci..

[57] Bigoni M (2021). Relationship between gait profile score and clinical assessments of gait in post-stroke patients. J. Rehabil. Med..

[58] Hsiao H, Zabielski TM, Palmer JA, Higginson JS, Binder-Macleod SA (2016). Evaluation of measurements of propulsion used to reflect changes in walking speed in individuals poststroke. J. Biomech..

[59] Hsiao H, Higginson JS, Binder-Macleod SA (2016). Baseline predictors of treatment gains in peak propulsive force in individuals poststroke. J. Neuroeng. Rehabil..

[60] Awad LN, Binder-Macleod SA, Pohlig RT, Reisman DS (2015). Paretic propulsion and trailing limb angle are key determinants of long-distance walking function after stroke. Neurorehabil. Neural Repair.

[61] Hsiao H, Awad LN, Palmer JA, Higginson JS, Binder-Macleod SA (2016). Contribution of paretic and nonparetic limb peak propulsive forces to changes in walking speed in individuals poststroke. Neurorehabil. Neural Repair.

[62] Patterson KK, Gage WH, Brooks D, Black SE, McIlroy WE (2010). Evaluation of gait symmetry after stroke: A comparison of current methods and recommendations for standardization. Gait Posture.

[63] Błazkiewicz M, Wiszomirska I, Wit A (2014). Comparison of four methods of calculating the symmetry of spatial-temporal parameters of gait. Acta Bioeng. Biomech..

[64] Tang A, Eng JJ, Rand D (2012). Relationship between perceived and measured changes in walking after stroke. J. Neurol. Phys. Ther..

[65] Perera S, Mody SH, Woodman RC, Studenski SA (2006). Meaningful change and responsiveness in common physical performance measures in older adults. J. Am. Geriatr. Soc..

[66] Zeilig G (2012). Safety and tolerance of the ReWalk™ exoskeleton suit for ambulation by people with complete spinal cord injury: A pilot study. J. Spinal Cord Med..

[67] Globas C (2012). Chronic stroke survivors benefit from high-intensity aerobic treadmill exercise: A randomized control trial. Neurorehabil. Neural Repair.

[68] Duncan P (1998). A randomized, controlled pilot study of a home-based exercise program for individuals with mild and moderate stroke. Stroke.

[69] Bellet RN, Adams L, Morris NR (2012). The 6-minute walk test in outpatient cardiac rehabilitation: Validity, reliability and responsiveness—A systematic review. Physiotherapy.

[70] Macchiavelli A, Giffone A, Ferrarello F, Paci M (2021). Reliability of the six-minute walk test in individuals with stroke: Systematic review and meta-analysis. Neurol. Sci..

[71] Shin SH, Lee MY (2014). Effect of gait training with additional weight on balance and gait in stroke patients. Phys. Ther. Rehabil. Sci..

[72] Roelker SA, Bowden MG, Kautz SA, Neptune RR (2019). Paretic propulsion as a measure of walking performance and functional motor recovery post-stroke: A review. Gait Posture.

[73] Nedergård H, Arumugam A, Sandlund M, Bråndal A, Häger CK (2021). Effect of robotic-assisted gait training on objective biomechanical measures of gait in persons post-stroke: A systematic review and meta-analysis. J. Neuroeng. Rehabil..

[74] McGrath R, Bodt B, Sergi F (2020). Robot-aided training of propulsion during walking: Effects of torque pulses applied to the hip and knee joints during stance. IEEE Trans. Neural Syst. Rehabil. Eng..

[75] Carollo JJ, Matthews D (2002). Strategies for clinical motion analysis based on functional decomposition of the gait cycle. Phys. Med. Rehabil. Clin. N. Am..

[76] Esquenazi A (2004). Evaluation and management of spastic gait in patients with traumatic brain injury. J. Head Trauma Rehabil..

[77] Fox MD, Delp SL (2010). Contributions of muscles and passive dynamics to swing initiation over a range of walking speeds. J. Biomech..

[78] Franks P, Bryan G, Martin R, Reyes R, Lakmazaheri A, Collins S (2021). Comparing optimized exoskeleton assistance of the hip, knee and ankle in single and multi-joint configurations. Wearable Technoligies.

[79] Saunders DH (2020). Physical fitness training for stroke patients. Cochrane Database Syst. Rev..

[80] Zhang J (2017). Human-in-the-loop optimization of exoskeleton assistance during walking. Science.

