# Supplementary material for: An impairment-specific hip exoskeleton assistance for gait training in subjects with acquired brain injury: a feasibility study
Source: Sci Rep. 2022 Nov 11;12:19343. doi: 10.1038/s41598-022-23283-w (PMC9652374; doi:10.1038/s41598-022-23283-w)
Supplement: Supplementary file 1 — Supplementary Information. [file 41598_2022_23283_MOESM1_ESM.docx]

# An impairment-specific hip exoskeleton assistance for gait training in subjects with acquired brain injury: a feasibility study

**Chiara Livolsi^1,2,*^, Roberto Conti^3^, Eleonora Guanziroli^4^, Þór Friðriksson^5^, Ásgeir Alexandersson^5^, Kristleifur Kristjánsson^5^, Alberto Esquenazi^6^, Raffaele Molino Lova^7^, Duane Romo^5^, Francesco Giovacchini^3^, Simona Crea^1,2,3,7^, Franco Molteni^4^, Nicola Vitiello^1,2,3,7^.**

**^1^** The BioRobotics Institute, Scuola Superiore Sant’Anna, Pontedera, Pisa, Italy

**^2^** Department of Excellence in Robotics & AI, Scuola Superiore Sant’Anna, Pisa, Italy

**^3^** IUVO S.r.l., Pontedera, Pisa, Italy

**^4^** Villa Beretta Rehabilitation Center, Valduce Hospital, Costa Masnaga, Lecco, Italy

**^5^** Össur, Reykjavík, Iceland

**^6^** Department of PM&R, MossRehab and Einstein Healthcare Network, Elkins Park, PA, USA

**^7^** IRCCS Fondazione Don Carlo Gnocchi ONLUS, Florence, Italy

**^*^** Corresponding author: chiara.livolsi@santannapisa.it

## Supplementary materials

Supplementary table I. Medical Research Council (MRC) strength grade for the hip and knee joints of the paretic limb.

|  | MRC strength grade | | | | | |  |
| --- | --- | --- | --- | --- | --- | --- | --- |
|  | Paretic hip | | | | Paretic knee | |  |
| ID | Hip flexors | Hip extensors | Hip abductors | Hip adductors | Knee flexors | Knee extensors | Group assignment |
| 1 | 4 | 4 | 3 | 3 | 4 | 4 | A |
| 2 | 4 | 4 | 4 | 4 | 4 | 4 | A |
| 3 | 3 | 3 | 3 | 3 | 3 | 3 | A |
| 4 | 3- | 2 | NA | NA | NA | 3- | A |
| 5 | 4- | 3- | 4 | 4 | 4- | 4 | A |
| 6 | 3- | 3- | 4 | 4 | 4 | 4 | A |
| 7 | 4 | 4 | 2 | 2 | 4 | 4 | B |
| 8 | 3 | 3 | 3 | 3 | 4 | 4 | B |
| 9 | 4 | 4 | 3 | 3 | 4 | 4 | B |
| 10 | 3 | 3 | 3 | 3 | 4 | 4 | B |
| 11 | 3 | 3 | 3 | 3 | 4 | 4 | B |
| 12 | 4 | 4 | 4 | 4 | 4 | 4 | B |
| 13 | 4 | 4 | 4 | 4 | 4 | 5 | B |
| 14 | 3- | 3- | 4 | 5 | 3 | 3- | B |

Legend: NA not available.

Supplementary table II. Modified Ashworth Scale (MAS) for the hip and knee joints of the paretic limb.

|  | MAS | | | | | |  |
| --- | --- | --- | --- | --- | --- | --- | --- |
|  | Paretic hip | | | | Paretic knee | |  |
| ID | Hip flexors | Hip extensors | Hip abductors | Hip adductors | Knee flexors | Knee extensors | Group assignment |
| 1 | 0 | 1 | 0 | 0 | 0 | 1 | A |
| 2 | 0 | 0 | 0 | 0 | 0 | 1 | A |
| 3 | 0 | 0 | 0 | 0 | 0 | 0 | A |
| 4 | 0 | 0 | 0 | 0 | 0 | 0 | A |
| 5 | 0 | 0 | 0 | 0 | 0 | 0 | A |
| 6 | 0 | 0 | 0 | 0 | 0 | 1+ | A |
| 7 | 0 | 0 | 0 | 0 | 1 | 0 | B |
| 8 | 0 | 0 | 2 | 0 | 1 | 1 | B |
| 9 | 0 | 1 | 0 | 0 | 0 | 1 | B |
| 10 | 0 | 0 | 0 | 0 | 0 | 1 | B |
| 11 | 0 | 0 | 0 | 0 | 0 | 0 | B |
| 12 | 0 | 0 | 0 | 0 | 0 | 0 | B |
| 13 | 0 | 0 | 0 | 0 | 0 | 0 | B |
| 14 | 0 | 0 | 0 | 0 | 0 | 1+ | B |


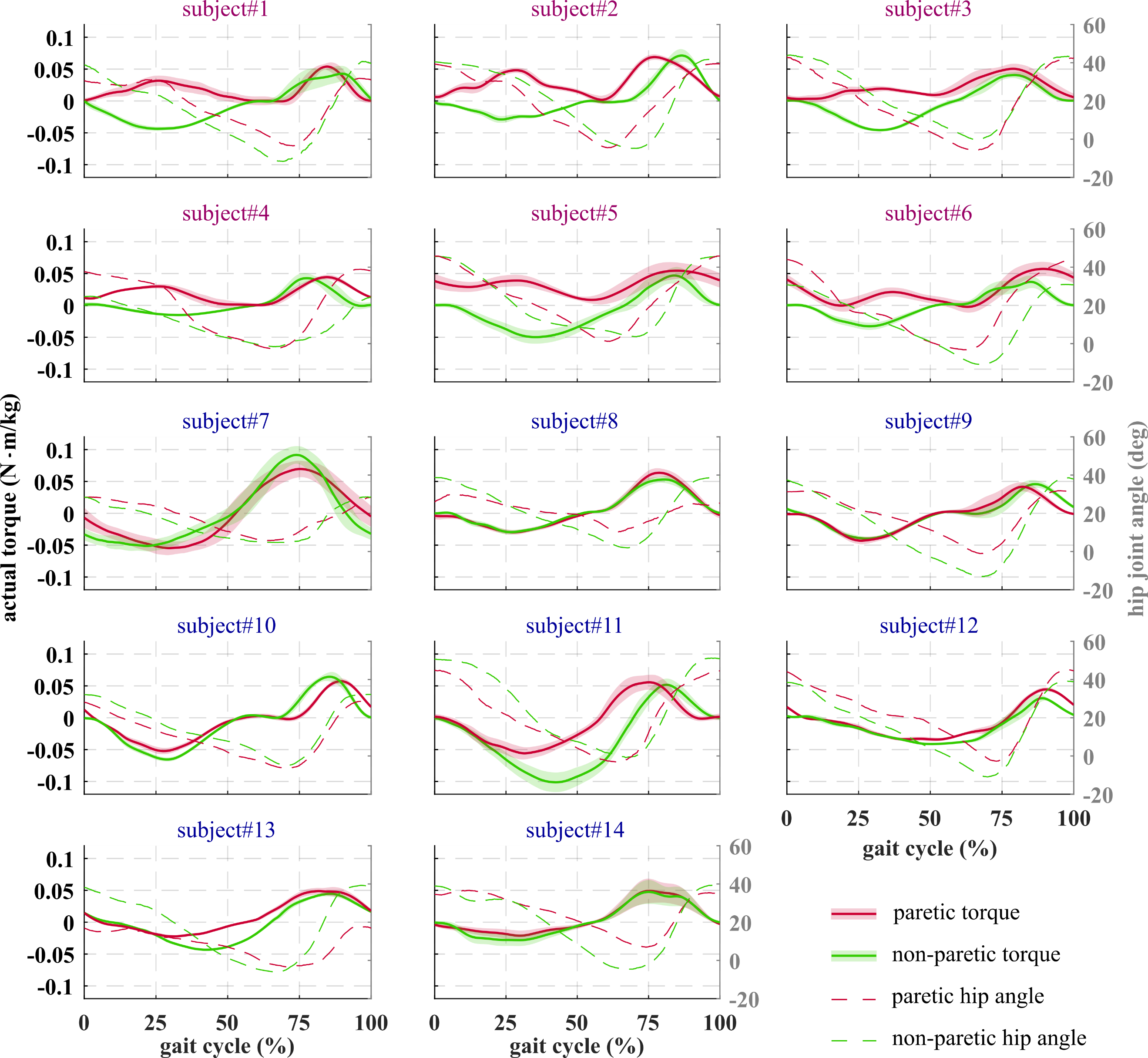


Supplementary figure 1. **Assistive profiles for the paretic and non-paretic sides of each study participant**. Average actual torque (solid lines) and hip joint angle (dashed lines) measured by the APO onboard sensors for the paretic (red) and non-paretic (green) sides are depicted for each study participant of group A (subject#1-6) and group B (subject#7-14). APO torque normalized to body mass and stride duration is represented as mean (solid lines) and standard deviation (shadow areas). The average profiles for each participant are computed over all strides in assistive mode of the 6MWT_APO_ at the end of the training.


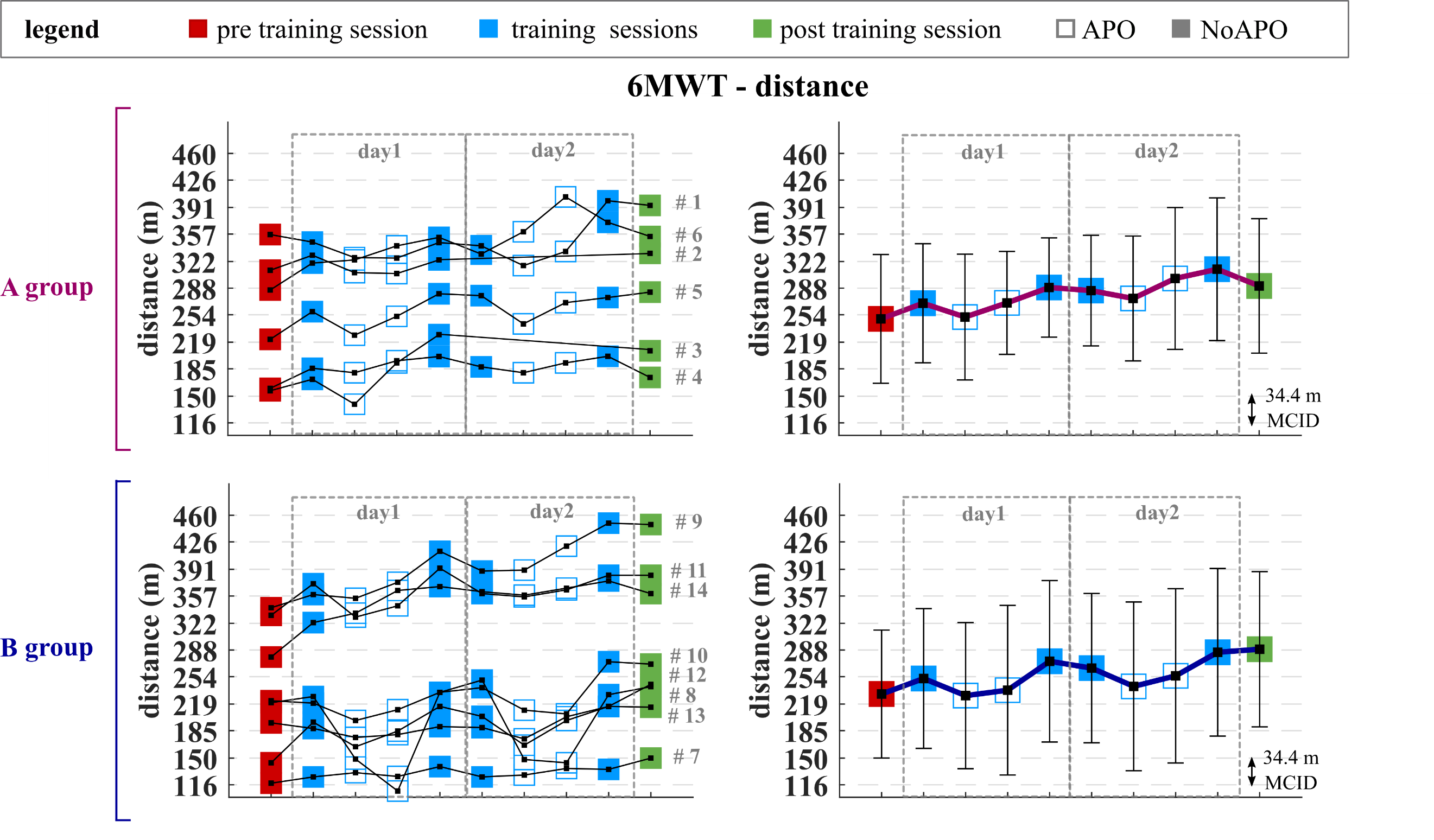


Supplementary figure 2. **Walked distances for each 6MWT performed throughout the study**. Data are reported individually (on the left) and grouped (top – A group, bottom – B group) as means and standard deviations (on the right). The minimal clinically important difference (MCID) is depicted in each plot with the dashed horizontal lines. Individual and group results are shown for each 6MWT of each session: pre and post training sessions without APO [NoAPO (red – pre training, green – post training)] and training sessions with and without the APO (blue) [NoAPO (full square) and APO (empty square)]. On the second training day (day 2), data of ID #2 and #3 (𝜖 A group) are missing because they performed only 1 training session due to their limited personal availability.

Supplementary table III. CR-10 Borg scale assessed after the 6MWTs (pre training NoAPO, end of training APO, post-training No APO). Last three rows represent the mean (standard deviation) of each study group (A, B) and all participants.

| ID | CR 10 – Borg scale | | |
| --- | --- | --- | --- |
|  | Pre training (No APO) | End Training  (APO) | Post training  (No APO) |
| #1 | 0 | 0 | 0 |
| #2 | 8 | 8 | 5 |
| #3 | 3 | 4 | 0 |
| #4 | 6 | 5 | 4 |
| #5 | 0 | 2 | 0 |
| #6 | 2 | 2 | 0.5 |
| #7 | 4 | 4 | 3 |
| #8 | 7 | 7 | 8 |
| #9 | 2 | 2 | 1 |
| #10 | 1 | 0 | 0 |
| #11 | 0 | 3 | 1 |
| #12 | 0 | 0 | 0 |
| #13 | 3 | 7 | 4 |
| #14 | 3 | 4 | 3 |
| A group (SD) | 3 (3) | 4 (3) | 2 (2) |
| B group (SD) | 3 (2) | 3 (3) | 3 (3) |
| All participants (SD) | 3 (3) | 3 (3) | 2 (2) |
